# Internal Jugular Vein Thrombosis in Isolated Tuberculous Cervical Lymphadenopathy

**DOI:** 10.1155/2016/5184196

**Published:** 2016-11-09

**Authors:** Sanjay Khaladkar, Avadhesh Chauhan, Arijit Ghosh, Kunaal Jain, Surbhi Chauhan

**Affiliations:** Department of Radio-Diagnosis, Dr. D. Y. Patil Medical College and Research Center, Pimpri, Pune, India

## Abstract

Tuberculosis is a common infectious disease with a high prevalence in developing countries and presents a major public health issue. Internal jugular vein (IJV) thrombosis is a rare complication in tuberculous cervical lymphadenopathy. We report a case of 26-year male patient with a history of low-grade evening rise in fever, dry cough, loss of appetite, and loss of weight with swelling in lower neck on right side. Ultrasonography (USG) neck showed well-defined hypoechoic lymph nodes posterior to right IJV and common carotid artery in the lower neck at level IV and in the right supraclavicular region showing central necrotic areas with adjoining IJV thrombosis. The association between tuberculosis and deep vein thrombosis is rare. Awareness of IJV thrombosis in isolated cervical lymphadenopathy needs high diagnostic suspicion and prompt treatment to avoid fatal complication. Our case is rare as there was isolated tuberculous cervical lymphadenopathy with adjoining IJV thrombosis. Both USG and computed tomography (CT) are accurate and reliable radiological investigations for detecting IJV thrombosis along with cervical lymph nodes. They are useful in assessing surrounding soft tissue and fat planes and knowing the size and extent of cervical lymphadenopathy. USG is inexpensive and readily available for monitoring response to treatment.

## 1. Introduction

IJV thrombosis is a rare condition. It is usually associated with central venous catheterization, trauma, neck surgery, coagulation disorders, hypercoagulation states, endocrine alteration, local infection or malignancy, occult malignancy, neck massage, deep neck infection, and intravenous drug abuse [1, 2]. Tuberculous cervical lymphadenopathy with IJV thrombosis is extremely rare complication of isolated tuberculous cervical lymphadenopathy [3].

## 2. Case Report

A 26-year male patient that presented with swelling in the right supraclavicular region since 1.5 months was referred by surgeon to ultrasound department for evaluation of neck swelling. There was a history of low-grade evening rise in fever, dry cough, loss of appetite, and loss of weight.

**Figure 1:**
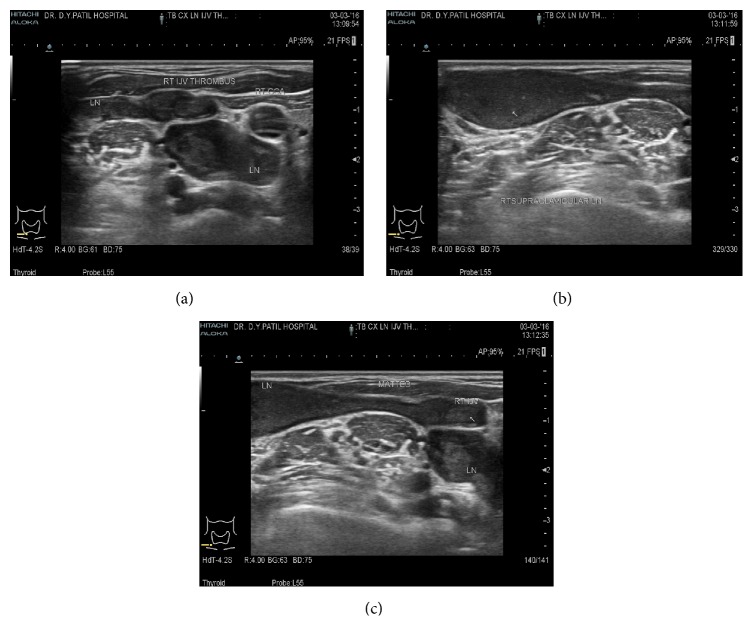
USG neck showing well-defined hypoechoic lymph nodes posterior to right CCA and IJV and in the right supraclavicular region at level IV with echogenic thrombus in right IJV.

**Figure 2:**
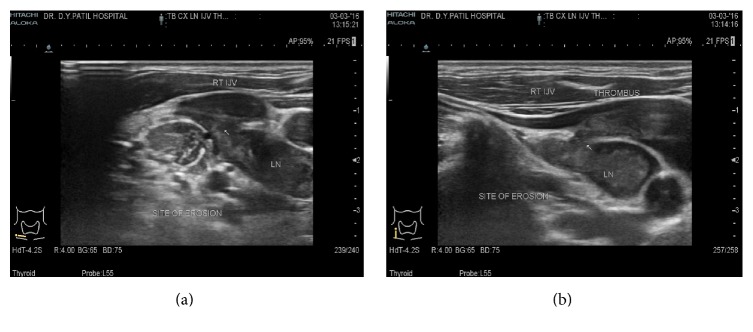
Cervical lymph node at level IV eroding through posterior and the right lateral wall of IJV with medium level echogenic IJV thrombus.

**Figure 3:**
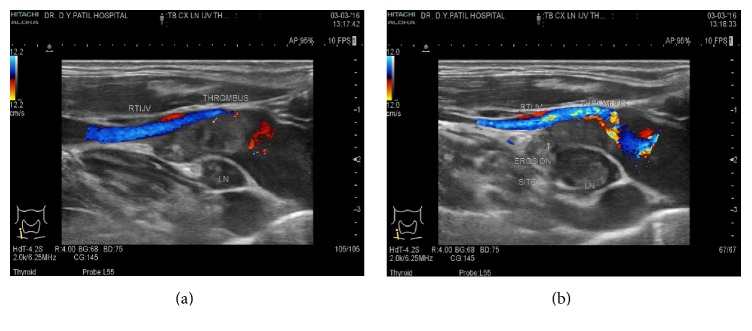
Color Doppler study showing IJV thrombus seen as filling defect.

**Figure 4:**
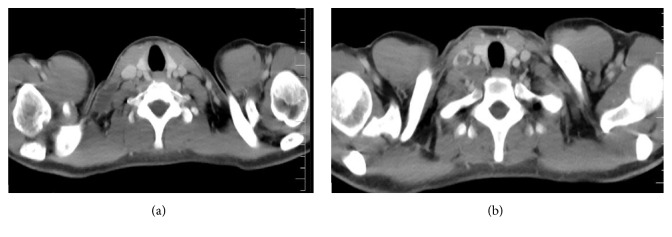
Axial CECT neck showing well-defined hypodense lymph node in the right supraclavicular region and posterior to right CCA and IJV at level IV showing peripheral rim enhancement with central caseation necrosis eroding through the posterior wall of right IJV with filling defect in contrast filled IJV lumen suggestive of thrombus.

**Figure 5:**
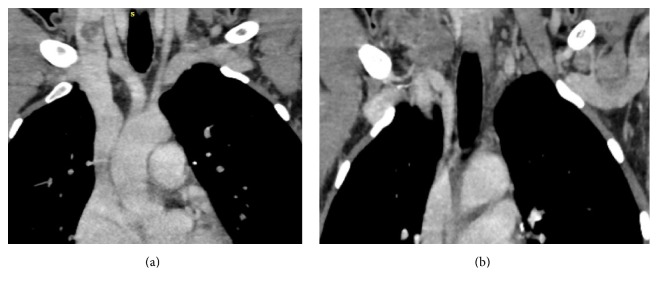
Coronal CECT neck showing right IJV thrombus and matted lymph nodes posterior to right CCA and IJV.

**Figure 6:**
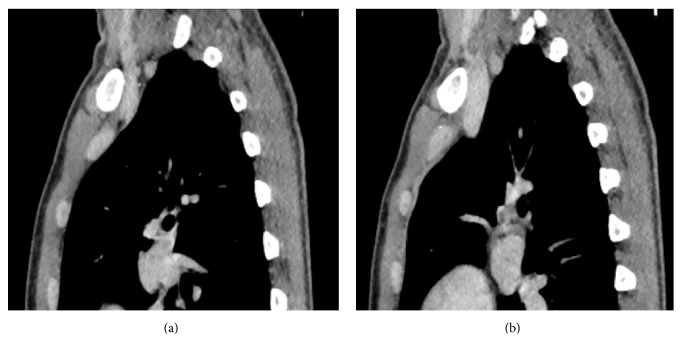
Sagittal CECT neck showing matted lymph nodes posterior to right IJV eroding through the posterior wall of right IJV with IJV thrombus.

USG neck showed well-defined hypoechoic lymph nodes measuring approx. 12 × 10 mm, 9 × 8 mm posterior to right IJV and common carotid artery (CCA) in the lower neck at level IV (Figures 1(a) and 1(b)). Lymph node measuring approx. 31 × 14 mm was noticed in the right supraclavicular region showing central hypoechoic areas due to necrosis showing multiple medium level internal echoes. A linear hypoechoic lesion of size approx. 20 × 5 mm was noted extending from this lymph node medially towards right lateral wall of internal jugular vein (Figure 1(c)). Echogenic fat plane noted between the lesion and right lateral wall of IJV was obscured with evidence of IJV invasion (Figures 2(a) and 2(b)). A medium level echogenic thrombus of size approx. 11(L) × 7(AP) × 8(T) mm was noted in adjoining right IJV in lower neck suggestive of IJV thrombosis (Figures 3(a) and 3(b)). Rest of the right IJV appeared normal with a normal flow on color Doppler. Contrast enhanced CT scan (CECT) of neck showed multiple lymph nodes at level IV on the right side and in the right supraclavicular region showing peripheral rim enhancement with central caseation necrosis (Figures 4, 5, and 6). A persistent filling defect was noted in right IJV in lower neck suggestive of IJV thrombosis (Figures 4, 5, and 6). Fine needle aspiration cytology (FNAC) of right supraclavicular swelling done after ultrasound and CECT neck revealed necrotizing granulomatous inflammation suggestive of tuberculosis. Patient was put on antituberculous treatment (ATT) along with anticoagulants after diagnosis since last one week and has been advised follow-up.

## 3. Discussion

Venous thrombosis results from disturbance of pathophysiological mechanisms in Virchow's triad of endothelial damage, stasis, and hypercoagulable state [1]. Complication of IJV thrombosis is septic emboli, intracranial venous sinus thrombosis, raised intracranial pressure, pulmonary embolism, facial edema, and loss of vision [1]. IJV thrombosis is uncommon and possibly life threatening complication. Early diagnosis is must for appropriate management, to prevent potentially fatal complication due to IJV thrombosis [1].

The associations between inflammation, hypercoagulable state, and hemostatic changes are established [4, 5]. Elevated plasma fibrinogen, impaired fibrinolysis with decreased level of antithrombin III, and reactive thrombocytosis favor development of DVT in pulmonary tuberculosis [6]. Thrombosis can also occur due to compression of the vein by lymph nodes in ganglionar form of TB as thrombosis can occur in the absence of any hemostatic abnormality [7]. Tuberculosis can cause thrombosis by various mechanisms like transitory hypercoagulable state, venous compression, and local invasion [4, 5, 7]. IJV thrombosis can occur from intracranial IJV to the junction of IJV with subclavian vein with the formation of brachiocephalic vein.

Tuberculous lymphadenopathy can cause extensive vessel wall damage with endothelial damage predisposing to thrombosis [3].

IJV thrombosis in our case is likely to be due to venous compression, local invasion with resultant endothelial damage, and transitory hypercoagulable state.

Both USG and CT are accurate and reliable radiological investigations for detecting IJV thrombosis, along with cervical lymph nodes. They are useful in assessing surrounding soft tissue and fat planes, and knowing the size and extent of cervical lymphadenopathy. USG is inexpensive and readily available for monitoring response to treatment. Both USG and CT scan show number and extent of cervical lymph nodes, central caseation necrosis, matted lymph nodes, IJV invasion, and IJV thrombus. Doppler and contrast enhanced CT scan shows patency of remaining IJV lumen. Early detection of IJV thrombosis is must to avoid complications like raised intracranial pressure, cerebral venous sinus thrombosis, pulmonary embolism, and septic emboli.

ATT should be immediately started in addition to anticoagulant therapy. The optimum duration of anticoagulation in IJV thrombosis is not yet standardized [3].

## 4. Conclusion

Tuberculous cervical lymphadenopathy is a rare cause of IJV thrombosis. Early detection with USG and/or CT is essential for timely treatment and to avoid further complications.
